# A Therapeutic Trial of Three Penicillins Conducted in a Casualty Department

**Published:** 1964-04

**Authors:** J. Woodyard, R. Hiles, W. R. G. Thomas, H. K. Bourns

**Affiliations:** Surgeon in Charge of Casualty Department, United Bristol Hospitals


### A THERAPEUTIC TRIAL OF THREE PENICILLINS CONDUCTED IN A CASUALTY DEPARTMENT

BY

J. WOODYARD, F.R.C.S., F.R.C.S. (E), R. HILES, M.B., W. R. G. THOMAS, M.B.
*Senior Casualty Officers*

AND

H. K. BOURNS, M.B., F.R.C.S.
*Surgeon in Charge of Casualty Department, United Bristol Hospitals*

### PENICILLIN TRIAL

There is at present considerable interest in the newer forms of penicillin which have been introduced since the isolation of the penicillin nucleus 6-amino penicillanic acid in 1959 made possible, by substitution of differing side chains, the production of a whole range of new penicillin compounds. This trial was designed to compare the clinical effectiveness of two of these, phenethicillin and propicillin with crystalline penicillin G given by injection. The antibiotic spectra of all three penicillins are comparable though not identical.

Propicillin is also known as P.A. 248 (Ultrapen) and B.R.L. 284 (Brocillin). Its chemical name is alpha phenoxypropyl penicillin potassium. Studies by Williamson *et al.* (1961) have shown it to be better absorbed after oral administration than phenethicillin or penicillin V, and they considered it to be the drug of choice in streptococcal infections. For penicillin-sensitive staphylococci all three were equally effective but against penicillin-resistant staphylococci propicillin was shown to be more effective *in vitro*, and since it gives the highest blood levels, it was felt that it would be least likely of the oral penicillins to permit the emergence of penicillin-resistant staphylococci. Jackson and Rao (1961) also showed that propicillin owed its effectiveness against penicillin-resistant staphylococci to its greater resistance to the hydrolytic effect of staphylococcal penicillinase on the penicillin molecule. Propicillin is active in the presence of protein, is non-toxic and apparently no more likely than other penicillins to produce hypersensitivity reactions. In addition it is acid-stable, producing adequate serum levels after oral administration, and its activity against Gram-positive cocci is comparable to penicillin V and phenethicillin. It is not effective against Gram-negative bacteria (Knox, 1962).

Studies of propicillin in general practice have shown its value in a wide variety of cases, including septic fingers and hands, infections of the respiratory tract, of the middle ear and skin (Schofield, 1962). The incidence of side-effects was negligible (Edgar, 1962).

The three penicillins used in the trial were:

(1) Crystalline penicillin in a dosage of 0·5 mega unit daily for three days;
(2) Propicillin in a dosage of 250 mgs by mouth four times a day for three days; and
(3) Phenethicillin in a dosage of 250 mgs by mouth four times a day for three days.

Each three-day period was considered to be one course of penicillin.

All patients who attended the casualty department of the Bristol Royal Infirmary and who were considered to require penicillin, automatically received one of the three penicillins. Choice of penicillin was random. The patient's index number was used as a key to a random list of the antibiotics. It was obviously impossible to hide the identity of the crystalline penicillin, but it was decided to conceal the identity of the other two. The actual type of penicillin was not known to the clinicians until the end of the trial.

The types of patient included in the trial were those with septic fingers and hand lesions, boils, abscesses, cellulitis and infected wounds. When a case was first seen an attempt was made not only to make an accurate diagnosis, but also to assess the stage of the infection. This is important because it has been shown in this department (Wesley James and Alder, 1961) that localization of an infection takes approximately the same length of time no matter what the virulence of the causative organism. A case with a six-days' history will probably have formed pus already so that delay in incision will produce a delay in treatment time (Table 14), as compared with a similar lesion seen on the second day in which suppuration might not yet have occurred. As soon as pus has formed, there are very few reasons for delaying the incision. In a toxic patient who has much oedema and cellulitis, an analgesic for twenty-four hours may be useful in order to allow him to rest, and an antibiotic in order to produce a therapeutic blood and tissue fluid level. Adequate anaesthesia is essential and to postpone a case until a skilled anaesthetist is available may often be very desirable.

The incision is important. The mismanagement of this simple step may alter the course of a straightforward lesion. Full exploration and the removal of a slough, if present, may be neglected by the inexperienced and this in itself will delay healing. In all cases the first dressing after incision was carried out 48 hours later unless the discharge was so profuse as to be likely to soak the dressings before this time had elapsed. All cases were seen to conclusion although in some cases discharge either to general practitioners or to clinics would have been both appropriate and desirable. Careful case histories were taken and these details together with details of the course of the conditions were eventually transferred to a record card for each patient. Special attention was paid to the date of discharge which was that on which the lesion had healed. The time from first attendance to discharge, reckoned in days, was known as the treatment time.

A swab was taken for culture as soon as pus was available. This was plated, in most cases, within six hours, the majority within three. Thus the identity of the pathogenic organism and its antibiotic sensitiveness was known within forty-eight hours of either the first attendance or of incision.

Occasionally the clinician considered that either a second course of the original antibiotic was indicated, or that a change of therapy to a non-penicillin antibiotic was necessary. This change was usually based on the result of the antibiotic sensitivities and on the clinical progress of the case. He was free to order such change in the therapy as he wished because the treatment of each individual case took precedence over all other considerations.

At the end of the trial a statistical analysis was carried out by means of hand-sorting the record cards and the construction of tables. They were then subjected to statistical analysis.

Methods used to assess the efficiency of each penicillin were:

(1) Comparison of treatment times, and

(2) Comparison of the incidence of complications.

The patients treated during the trial were seen by four separate clinicians and so individual opinions might possibly have affected the judgement of each case. One of us (H.K.B.) was present all the time and the other three had some previous experience of septic conditions, and there was always an overlap when one took over from the other. All clinicians were carefully briefed in the rules by which the trial was conducted, and as many variables as possible were eliminated. The length of the trial was limited so that all the staff in the hospital, such as the nurses who performed the dressings, should not change completely during its course, and so that interest should not flag.

### THE RESULTS OF THE TRIAL

When the record cards were being completed for each patient it became evident that it was often not possible to assess the treatment time since the patients had not returned for discharge. Thus of the 495 patients commencing their course of penicillin, 72 had to be rejected from the trial.

Table 1 shows the number of rejected patients (termed defaulters) in each group.

TABLE 1

| | Crystalline penicillin | Propicillin | Phenethicillin | Totals |
|---|---|---|---|---|
| Full attendance | 135 | 132 | 156 | 423 |
| Defaulters | 27 (16·7%) | 25 (15·9%) | 20 (11·4%) | 72 (14·5%) |

Total number of patients commencing the trial = 495.

From the table it can be seen that the percentage of defaulters in each group was not significantly different. Very close supervision of oral administration is important. Patients must be carefully instructed and equally carefully questioned about the tablets, because many will not admit to lapse of memory or such like unless they realize that one will not be put off by a vague answer. We found that a very good method of checking this point was to find out how many tablets were left at any visit. This would of course give the answer as to the correctness of self administration. Verbal and written instructions were given, but constant reminders were necessary in order that the dosage should be accurately maintained even for such a short period as three days. Injections were given by a nurse on the instructions of a doctor and so this form of administration did not depend on the vagaries of the patient. There were, however, defaulters in all groups, and the effect of the wish to escape injections on the one hand and the omission to take tablets on the other cancelled each other out (Tables 1 and 6).

Of the 423 patients who attended throughout the trial, 10 had a further course of penicillin and 14 a further course of a non-penicillin antibiotic. In most cases this was either tetracycline or streptomycin.

TABLE 2

| | Crystalline penicillin | Propicillin | Phenethicillin | Totals |
|---|---|---|---|---|
| 1 course of penicillin | 128 | 122 | 149 | 399 |
| 2 courses of penicillin | 1 | 5 | 4 | 10 |
| Change to NON-penicillin antibiotic | 6 | 5 | 3 | 14 |

It can be seen in Table 2 that numbers are generally comparable, except that only one patient received a further course of intra-muscular penicillin. This was possibly due to reluctance on the part of the clinician to subject patients to a course of a further three injections. Patients receiving a repeat course of penicillin or a change to another antibiotic were rejected from further statistical analysis. This was thus performed on the results of treatment of the remaining 399 patients.

### ANALYSIS OF TREATMENT TIMES

The patients were divided into three groups based on their treatment times (Tables 3 and 4). The 399 cases were also subjected to a bacteriological classification. The bacteriological groups were:

1. Patients whose infection was due to penicillin-sensitive staphylococcus aureus.
2. Cases where the pathogenic organism was a penicillin-resistant staphylococcus aureus.
3. Cases where the pathogenic organisms were two strains of staphylococcus aureus, the one penicillin-sensitive and the other penicillin-resistant.
4. Cases where no bacteriological studies were carried out due to the absence of pus; these were mainly cases of cellulitis and cases of minor intra-dermal infections.
5. Cases where no bacteriological studies were carried out despite the presence of pus. This group contained patients whose bacteriological swabs grew no organisms—but also cases where no swab was taken due to neglect on the part of the clinician.
6. Patients whose infections were due to non-staphylococcal bacteria, e.g. B. coli and streptococci.

Table 3 shows the numbers of patients in these categories produced by classifying the patients into bacteriological groups (the horizontal columns) and into the three treatment time groups, i.e. 0–6, 7–12 and 13+ days. These are the vertical columns. *T* denotes the total in each bacteriological group for each penicillin.

TABLE 3

| | Crystalline penicillin | | | | Propicillin | | | | Phenethicillin | | | |
|---|---|---|---|---|---|---|---|---|---|---|---|---|
| | 0–6 | 7–12 | 13+ | T | 0–6 | 7–12 | 13+ | T | 0–6 | 7–12 | 13+ | T |
| Infections by penicillin sensitive *S. aureus* | 19 | 25 | 4 | 48 | 17 | 26 | 3 | 46 | 17 | 22 | 10 | 49 |
| Infections by penicillin resistant *S. aureus* | 8 | 9 | 2 | 19 | 9 | 9 | 1 | 19 | 6 | 5 | 5 | 16 |
| Infections by mixed growth of penicillin sensitive and resistant organisms | 1 | 2 | — | 3 | — | — | — | — | — | — | — | — |
| No bacteriology due to no pus | 19 | 13 | — | 32 | 20 | 11 | 3 | 34 | 29 | 12 | 3 | 44 |
| No bacteriology but pus present | 8 | 8 | — | 16 | 8 | 5 | 1 | 14 | 14 | 10 | 1 | 25 |
| Infections due to other organisms | 7 | 2 | 1 | 10 | 4 | 4 | 1 | 9 | 9 | 4 | 2 | 15 |
| TOTALS | 62 | 59 | 7 | 128 | 58 | 55 | 9 | 122 | 75 | 53 | 21 | 149 |

Table 3 also demonstrates that there was no significant difference between the results of treatment in the three groups. All three forms of therapy proved equally effective and the results clinically indistinguishable.

*Incidence of complications*

Table 4 shows the percentage incidence of straightforward and complicated cases in the three groups of patients. All these patients had only one course of penicillin.

TABLE 4

| | Straightforward | Complicated | Total per cent | Number of patients |
|---|---|---|---|---|
| Crystalline penicillin | 85·9 | 14·1 | 100 | 128 |
| Propicillin | 85·2 | 14·8 | 100 | 122 |
| Phenethicillin | 85·2 | 14·8 | 100 | 149 |

Table 4 also demonstrates that the incidence of complications is even. The characteristics of these complications were:

(1) Prolonged healing for no apparent reason;
(2) relapse or the development of new lesions.
(3) development of sloughs;
(4) development of osteitis;
(5) development of pus during therapy;
(6) residual disability; and
(7) side effects of treatment.

The individual numbers of complicated cases were so small and their distribution in the various categories of patients so even that individual tables showing the distributions are not shown.

### CONCLUSION

So far it was impossible to demonstrate any real difference between the three penicillins. It was considered that this might have been due to an insufficient dosage of penicillin. The trial was therefore repeated but with double the dosage of penicillin. Only two antibiotics were used so enabling an approximately similar number of cases to be collected in a shorter time. It also enabled us to compare the results of the oral and intramuscular method of treatment.

### THE SECOND PART OF TRIAL

Two penicillins were used in the second part.

1. Crystalline penicillin in a dosage of 1 mega unit daily for 3 days.
2. Propicillin in a dosage of 500 mgs four times a day for 3 days.

Table 5 shows the numbers of patients who attended for the full course of treatment and also the defaulters. The total number of patients who started in the trial was 583; the defaulters were rejected.

TABLE 5

| | Crystalline penicillin | Propicillin | Totals |
|---|---|---|---|
| Full attendances | 224 | 241 | 465 |
| Defaulters | 66 | 52 | 118 |

Table 6 shows the number of patients in each therapeutic group who needed a second course of penicillin or a change to a "non-penicillin" antibiotic.

TABLE 6

| | Crystalline penicillin | Propicillin | Totals |
|---|---|---|---|
| 1 course of penicillin | 187 | 205 | 392 |
| 2 courses of penicillin | 9 | 19 | 28 |
| Change to non-penicillin antibiotic | 28 | 17 | 45 |

Table 7 shows the number of patients in each bacteriological group.

TABLE 7

| | Crystalline penicillin | Propicillin |
|---|---|---|
| Penicillin-sensitive *S. aureus* infections | 65 | 71 |
| Penicillin-resistant *S. aureus* infections | 26 | 34 |
| Infections due to *S. aureus* with penicillin-sensitive and resistant strains | — | 1 |
| Absence of pus | 32 | 38 |
| No bacteriological studies but pus present | 42 | 46 |
| Infections due to other bacteria | 22 | 15 |
| TOTALS | 187 | 205 |

An attempt to compare treatment time by dividing the patients into three groups as shown in Table 3 was made, but it is open to criticism since it is a gross method of comparing treatment times and does not take into account the wide spread of cases in each group. Construction of tables to present the statistics is difficult. Three columns of figures are needed for each type of penicillin before further subdivision is attempted. The number of cases in each group then becomes so small that comparison becomes difficult and conclusions of doubtful value.

It was therefore decided to obtain the advice of a statistician who suggested that analysis could be better performed by calculating the average treatment time for each group, noting the difference, and comparing this to the least significant difference (L.S.D.). This was carried out for the second part of the trial where the dosage of crystalline penicillin was 1 mega unit and that of propicillin 500 mg four times per day.

It was decided to include cases which have received a repeat course of antibiotic and who have also received a course of non-penicillin antibiotic during their illness.

The variate used for comparison was the mean number of days on treatment, and the tables show means for each treatment, their difference and their least significant difference at the P = 0·05 level. To make some allowance for the differential effects of the treatments in different categories of cases, the means are given for various one-way groupings of the patients.

Table 10 gives the overall means in treatment groups, unadjusted for any difference in composition of the groups. The mean difference in treatment time, 1·3 days shorter for propicillin, was very nearly equal to its least significant value, 1·4 days. There was thus some evidence of a real overall advantage of propicillin, but the least significant difference was not quite small enough to show the differences as formally significant of the P = 0·05 level.

Table 8 shows the incidence of complicated cases in each of the two therapeutic groups. These cases had only one course of antibiotic.

TABLE 8

| | Straightforward | Complicated | Total per cent | Total No. of patients |
|---|---|---|---|---|
| Crystalline penicillin | 88·8 | 11·2 | 100 | 187 |
| Propicillin | 94·1 | 5·9 | 100 | 205 |

Table 9 shows the incidence of complicated cases in each of the three therapeutic groups—subdivided into the three main bacteriological classifications. This shows that incidence of complications is less with propicillin when the pathogenic organism

TABLE 9

| | Straightforward | Complications | Total | Number of patients |
|---|---|---|---|---|
| Infections due to penicillin-sensitive *S. aureus* | | | | |
| Crystalline penicillin | 95·4 | 4·6 | 100 | 65 |
| Propicillin | 94·4 | 5·6 | 100 | 71 |
| Infections due to penicillin-resistant *S. aureus* | | | | |
| Crystalline penicillin | 73·1 | 26·9 | 100 | 26 |
| Propicillin | 91·2 | 8·8 | 100 | 34 |
| Infections where no bacteriological studies were carried out due to absence of pus | | | | |
| Crystalline penicillin | 84·4 | 15·6 | 100 | 32 |
| Propicillin | 97·4 | 2·6 | 100 | 38 |

is a penicillin-resistant S. aureus, and when the case has no pus available for culture. With penicillin-sensitive Staph. aureus infections, the incidence of complicated cases was similar in the two groups.

Tests of statistical significance were applied to these results and in cases of infection due to penicillin-resistant Staph. aureus infections and in cases with no pus, the conclusions approached statistical significance.

### CONCLUSION

Increasing the dosage of propicillin and crystalline penicillin showed the superiority of propicillin when treating cases which did not produce any pus. These are mainly cases of cellulitis. No real difference could be shown between the two penicillins when treating Staph. aureus infections.

It is of interest that, in the first part of the trial, the incidence of penicillin-resistant *Staphylococcal aureus* infections was 38 per cent and in the second phase with increased dosage, 42 per cent. In 1961 the percentage of penicillin-resistant staphylococci was 29.

Table 10 demonstrates the comparison of average treatment times in days, for patients who received crystalline penicillin and for those who received propicillin. All patients are included in this table. The total number of patients is given in parenthesis. This shows an apparent superiority of propicillin, the difference in average treatment times approaching the least significant difference.

TABLE 10

| Average treatment times for crystalline penicillin | Average treatment times for propicillin | Difference | L.S.D. ($P = 0{\cdot}05$) |
|---|---|---|---|
| 11·0 (224) | 9·7 (241) | −1·3 | 1·4 |

Table 11 shows average treatment time in days for crystalline penicillin and propicillin all the patients being subjected to a classification based on diagnosis.

TABLE 11

| Diagnosis | Treatment times for crystalline penicillin | Treatment times for propicillin | Difference | L.S.D. ($P = 0{\cdot}05$) |
|---|---|---|---|---|
| Paronychia | 7·8 (43) | 8·3 (45) | 0·5 | 1·6 |
| Carbuncle | 22·0 (3) | 17·3 (3) | − 4·7 | 28·0 |
| Boil | 11·4 (43) | 11·4 (38) | 0 | 2·9 |
| Abscess | 11·5 (73) | 11·2 (79) | − 0·3 | 2·6 |
| Cellulitis | 12·3 (23) | 6·1 (23) | − 6·2* | 5·9 |
| Intradermal infection | 11·1 (36) | 8·6 (51) | − 2·5 | 3·4 |
| Other | 20·3 (3) | 7·5 (2) | −12·8 | 20·6 |

* Indicates that the difference was significantly less than zero at the least $P = 0{\cdot}05$ level.

With all these different diagnoses propicillin produced shorter treatment times, with the exception of boils and paronychia. It was, however, only with cases of cellulitis that the difference in the mean treatment times exceeded the least significant difference.

Table 12 shows average treatment times for the two penicillins, the patients being subject to a bacteriological classification.

TABLE 12

| | Average treatment time for | | Difference | L.S.D. ($P = 0{\cdot}05$) |
|---|---|---|---|---|
| | Crystalline penicillin | Propicillin | | |
| Penicillin-sensitive *S. aureus* | 9.0 (72) | 9·7 (81) | +0·7 | 1·5 |
| Penicillin-resistant *S. aureus* | 13·5 (36) | 13·1 (51) | −0·4 | 4·2 |
| Penicillin-sensitive and resistant *S. aureus* | 16–0 (2) | 7·0 (1) | −9·0 | 176·1 |
| No bacteriology due to no pus | 9·6 (35) | 6·5 (48) | −3·1* | 2·6 |
| No bacteriology but pus present | 10·4 (51) | 8·3 (48) | −2·1 | 2·4 |
| Infections due to other organisms | 15·7 (28) | 11·3 (18) | −4·4 | 7·4 |

* Indicates a significant difference at at least the $P = 0{\cdot}05$ level.

Crystalline penicillin produced a shorter treatment time with infections due to penicillin-sensitive S. aureus—but the least significant difference was not exceeded. With penicillin-resistant S. aureus infections, cases where no bacteriology was carried out due to absence of pus (mainly cases of cellulitis) and where pus was present but bacteriological studies neglected—propicillin produced a shorter average treatment time but it was only in patients with no pus that the difference exceeded the least significant difference.

It can be seen from Table 12 that cases with infections due to penicillin-resistant organisms took longer to heal than those with infections due to penicillin-sensitive staphylococci, whichever penicillin was used. Penicillin is of little value once pus has formed in that it has no direct effect on the fate of healing (Anderson 1958), but it does reduce the size of the lesion and in this way reduces the dimensions of the cavity that has to be filled in. Thus the real value of penicillin is obtained when it is given early in adequate dosage. There must be many cases seen and treated early by the general practitioners. These never reach hospital because the lesion may be aborted before pus has formed. The trouble arises when antibiotic therapy is continued long after pus has formed, thus leading to delay in incision (Table 14).

Table 13 shows the average treatment times for the two penicillins, the patients being further subdivided into four groups according to the treatment the patient received prior to being seen in the casualty department.

The groups used were:

(1) Patients who had received no treatment.

(2) Those who had previously received penicillin from their own general practitioner.

(3) Those who had received an antibiotic other than penicillin from their own G.P.; and

(4) Those who had received local topical treatment usually poulticing or application of antiseptic lotion from the G.P. or self-administered.

TABLE 13

| | Crystalline penicillin | Propicillin | Difference | L.S.D. ($P = 0{\cdot}05$) |
|---|---|---|---|---|
| No previous treatment | 11·0 (175) | 0·4 (209) | −1·6* | 1·5 |
| Previous penicillin | 11·7 (19) | 11·2 (20) | −0·5 | 4·2 |
| Previous non-penicillin antibiotic | 10·3 (10) | 9·8 (4) | −0·5 | 7·6 |
| Previous topical treatment | 11·4 (20) | 13·5 (8) | +2·1 | 7·9 |

* Indicates a significant difference at at least the $P = 0{\cdot}05$ level.

In the first three groups propicillin produced shorter treatment times but it was only in the group which had not received any previous therapy that the difference exceeded the least significant difference. The last three groups contain so few cases that the results are not very helpful.

TABLE 14

| | Crystalline penicillin | Propicillin | Difference | L.S.D. ($P = 0{\cdot}05$) |
|---|---|---|---|---|
| No surgery performed | 10·0 (59) | 7·5 (77) | −2·5* | 2·4 |
| Surgery on 1st and 2nd days | 10·6 (142) | 10·4 (139) | −0·2 | 1·6 |
| Surgery on 3rd or later days | 16·8 (23) | 12·0 (25) | −4·8 | 6·0 |

* Indicates a significant difference at at least the $P = 0{\cdot}05$ level.

Table 14 shows the average treatment time in days in relation to the incidence of surgical treatment. This table demonstrates that those cases which required surgery took longer to heal than cases which did not need it. This is what one would expect. Of those in which an operation was performed, the longer this was delayed, the longer they took to heal. This fact supports the point that has already been made that delay in surgery will always lead to a delay in healing time. It can be seen from this table that propicillin produced shorter average treatment times in all three groups, but only in the group upon which surgery was not performed was the reduction in treatment time significantly shorter. These cases correspond to those in the "no bacteriological studies due to absence of pus" group (Table 12) which were often cases of cellulitis.

### CONCLUSIONS

A comparison of the average treatment times confirms the superiority of propicillin in cases where no pus was available for culture. These were usually cases of cellulitis, but some were probably staphylococcal infections in which healing by resolution was achieved. No real difference between propicillin and crystalline penicillin is established for infections caused by *Staphylococcus aureus* whether penicillin-sensitive or not.

Many of the tables show results which do not demonstrate any statistically significant superiority of one form of penicillin over another, but they do indicate that propicillin is at least the equal of the other types of penicillin under examination.

They also show that the oral method of administration of penicillin is as efficacious as the intramuscular route for the type of cases under review.

### SUMMARY

A comparative trial of oral and systemic penicillin was conducted in a Hospital Casualty department. In the first part of the trial penicillin G was compared with phenethicillin and propicillin. No significant difference was noted between the three forms of treatment. In a further study the doses were doubled and the effect of one mega unit of benzyl penicillin given daily was compared with 500 mgs of propicillin given four times a day. Results showed that in the treatment of cellulitis the response to propicillin was considerably better and that the advantage was statistically significant. With the other infections, a slight advantage of propicillin was demonstrated but this was not significant.

*Acknowledgements*

We are indebted to Pfizer Limited who supplied the propicillin and phenethicillin for both parts of the trial. We are grateful to their medical advisers and in particular to Dr. J. Goulton and to Mr. P. R. D. Avis, a statistician, who kindly carried out the final analysis of the results. We are very grateful for the untiring devotion of Sister Doyle and Mr. Stevens and of all the Nurses of the Casualty Department of the Bristol Royal Infirmary. Finally we thank the Board of the United Bristol Hospitals who made the whole thing possible by paying the salary of an extra Casualty Officer for the duration of the trial.

### REFERENCES

Anderson, J. (1958). *Brit. med. J.*, **2,** 1569.
Edgar, J. H. (1962). *Practitioner*, **189,** 212.
Jackson, F. L. and Rao, K. K. (1961). *Lancet*, **1,** 850.
Knox, R. (1962). *Nature*, **192,** 492.
Schofield, P. B. (1962). *Practitioner*, **188,** 115.
Wesley-James, O. and Alder, V. G. (1961). *J. Clin. Path.*, **14,** 96.
Williamson, G. M. *et al.* (1961). *Lancet*, **1,** 847.
G.P. Research Group (1962). *Practitioner*, **188,** 125.

